# Vissage percutané rétrograde du scaphoïde

**DOI:** 10.11604/pamj.2016.23.83.8799

**Published:** 2016-03-11

**Authors:** Younes Mhammdi, Berrada Mohamed Saleh

**Affiliations:** 1 Service de Traumatologie Orthopédie, Hopital Ibn Sina, CHU Rabat, Maroc

**Keywords:** Scaphoïde, vissage percutané rétrograde, fracture, Scaphoid, retrograde percutaneous screwing

## Image en medicine

En plus des bénéfices de la chirurgie à ciel ouvert du scaphoïde, la réduction de la durée d'immobilisation et le retour rapide au travail, le vissage percutané permet également de réduire le risque opératoire et d’éviter un délabrement des tissus mous, une dévascularisation du foyer de fracture et par conséquent cette technique présente moins de risque de pseudarthrose. Les auteurs rapportent le cas d'un patient de 27 ans, opéré pour une fracture non déplacée type 4 de Schernbereg. Il a bénéficié d'un vissage percutané rétrograde. Les suites étaient simples, et la sortie à J1 post-opératoire avec une orthèse du poignet, que le patient a gardé pendant 15 j, puis il a commencé l'auto rééducation. La consolidation a été obtenue vers 2 mois et 15j, il s'agit d'un étudiant qui n'as pas arrêté ses cours. Cependant, on lui a interdit tout travail de force pendant 3 mois. Il s'agit d'une technique qui donne de très bon résultats dans l'immédiat, son indication est limitée aux fractures non déplacées du scaphoïde.

**Figure 1:**
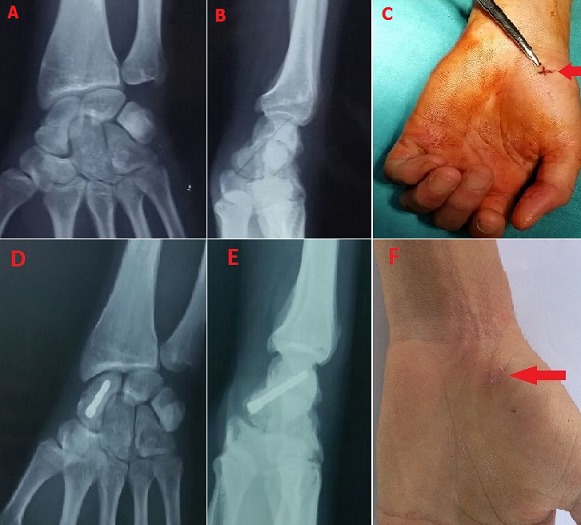
(A) radio préopératoire de face montrant une fracture non déplacée du scaphoïde; (B) radio préopératoire de profil montrant une fracture non déplacée du scaphoïde; (C) image peropératoire montrant l'abord de 5mm ayant permis le visage; (D) radio de contrôle post-opératoire de face montrant la stabilisation de la fracture par une vis d'HERBERT; (E) radio de contrôle post-opératoire de face montrant la stabilisation de la fracture par une vis d'HERBERT; (F) aspect du poignet et de la cicatrice opératoire 2 semaines après l'intervention chirurgicale

